# Quantitative Proteomics and Metabolomics Reveal Biomarkers of Disease as Potential Immunotherapy Targets and Indicators of Therapeutic Efficacy

**DOI:** 10.7150/thno.37373

**Published:** 2019-10-15

**Authors:** Melanie A. MacMullan, Zachary S. Dunn, Nicholas A. Graham, Lili Yang, Pin Wang

**Affiliations:** 1 Mork Family Department of Chemical Engineering and Materials Science, University of Southern California, Los Angeles, California; 2 Department of Microbiology, Immunology & Molecular Genetics, University of California, Los Angeles, California; 3 Eli & Edythe Broad Center of Regenerative Medicine and Stem Cell Research, University of California, Los Angeles, California; 4 Department of Biomedical Engineering, University of Southern California, Los Angeles, California; 5 Department of Pharmacology and Pharmaceutical Sciences, University of Southern California, Los Angeles, California

**Keywords:** Quantitative mass spectrometry, immunotherapy targets, proteomics, metabolomics

## Abstract

Quantitative mass spectrometry (MS) continues to deepen our understanding of the immune system, quickly becoming the gold standard for obtaining high-throughput, quantitative data on biomolecules. The development of targeted and multiplexed assays for biomarker quantification makes MS an attractive tool both for diagnosing diseases and for quantifying the effects of immunotherapeutics. Because of its accuracy, the use of MS for identifying biomarkers of disease reduces the potential for misdiagnosis and overtreatment. Advances in workflows for sample processing have drastically reduced processing time and complexities due to sample preparation, making MS a more accessible technology. In this review, we present how recent developments in proteomics and metabolomics make MS an essential component of enhancing and monitoring the efficacy of immunotherapeutic treatments.

## Introduction

**Figure 1:**
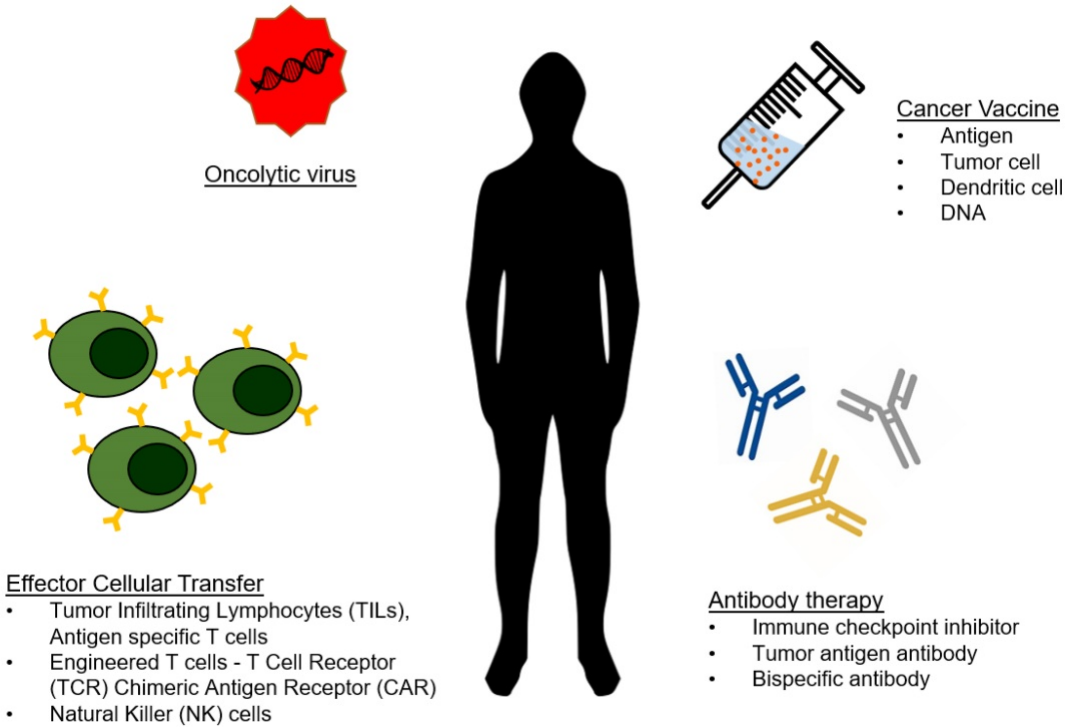
** Type of cancer immunotherapy.** The arsenal of cancer immunotherapy has grown to feature four main types. Effector cellular transfer can utilize lymphocytes gathered from tumor tissue (TILs) or cultured with tumor antigens (antigen specific T cells), or genetically modified lymphocytes. Oncolytic viruses are viruses that preferentially target and kill cancer cells, and are more recently being engineered to introduce immuno-stimulating transgenes. Cancer vaccines prime the immune system to eliminate cancer from the recognition of cancer antigens. Antibody therapies include direct targeting of tumor antigens, as well as by stimulating an antitumor response by blocking inhibitory signals and/or providing additional stimulation.

As analytical technologies continue to become faster, smaller, and less expensive, we are moving towards an era of personalized medicine. Because of this, engineers can focus on developing the body's own machinery and defense mechanisms as therapeutics, contributing to the growing field of immunotherapy. To date, a wide range of immunotherapeutics are being developed that capitalize on various aspects of interactions between diseased cells and immune cells. The most popular and successful immunotherapies for cancer have been checkpoint inhibitors and genetically engineered T-cells and antibodies, but many alternative forms of immunotherapy have been explored (Figure 1) 1-4.

**Figure 2:**
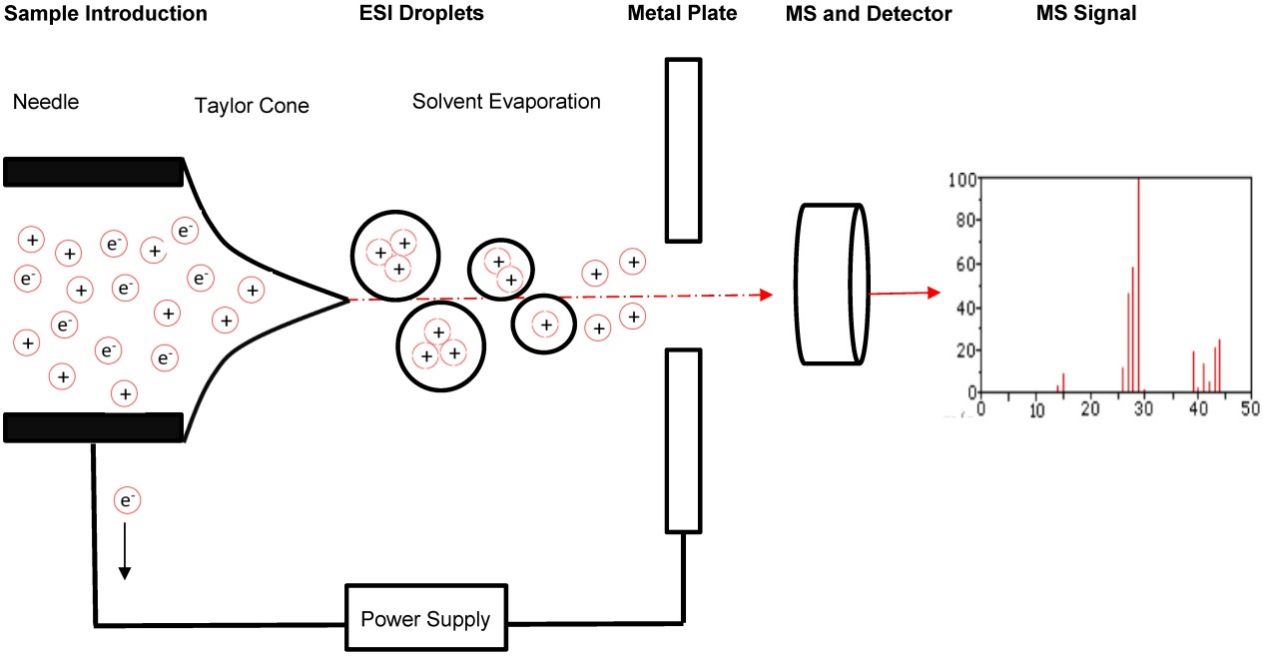
** Schematic of sample flow through MS.** A typical and general mass spectrometer set-up includes the introduction of a sample through an electrospray ionization source to produce protonated molecules. These molecules are then directed through an electromagnetic field to facilitate separation by mass-to-charge (m/z) ratio. A detector records information about the molecules passing through the mass analyzer that can be evaluated further through comparison to molecular mass libraries.

Advances in immunotherapy have been met with a growing need for theranostic tools, sensitive diagnostic devices which can evaluate the efficacy of these immunotherapies and provide guided strategies for improvement. A growing body of research suggests that one technology is optimal for this purpose: the mass spectrometer 5, 6. Mass spectrometers are used in proteomics to analyze small fragments of proteins, called peptides. Through the use of electromagnetic fields, mass spectrometers separate peptides in a sample based on mass-to-charge (m/z) ratio and then quantify peptide levels and analyze their characteristics 7 (Figure 2). They do so with high accuracy and sensitivity, enabling identification of minute differences in the expression of a peptide across cell conditions. The interaction between host cells and immune cells results when antigen-presenting cells (APCs) display internally developed peptides on their surface via major histocompatibility complex (MHC) molecules 8. The peptides that elicit an immune response are called antigens, and the specific portion of the antigens recognized by antibodies, B cells, or T cells are known as epitopes. As a result of cellular stress and disease, mutated or foreign peptides are presented by cells, and when a peptide is mutated or foreign it often has a different m/z ratio than that of peptides normally present in cells, enabling detection by mass spectrometry 9. Furthermore, in response to disease and disease treatment, the expression level of peptides changes, which can be monitored by mass spectrometry. Thus, foreign or differentially expressed antigens on a cell surface can be indicative of a disease or of a therapeutic response, and we can classify these peptides as biomarkers of disease if they can be identified and characterized.

A biomarker is defined as “a characteristic that is objectively measured and evaluated as an indicator of normal biological processes, pathogenic processes, or pharmacologic responses to a therapeutic intervention” 10, 11. Mass spectrometers have the capacity to aid in both the discovery of biomarkers and in the validation and verification of those biomarkers in disease. The multiple types of mass spectrometers that are popular for immunotherapy assessment have varying degrees of sensitivity and specificity 12. In this review, we describe some of the popular varieties and discuss how the mass spectrometer has become an essential theranostic tool for the field of immunotherapy, aiding in the understanding of the immune system and helping to improve and evaluate immunotherapies.

To date, there are two commonly used ion sources and five commonly used mass analyzers that can be combined to yield a mass spectrometer ideal for a wide range of proteomic analyses 13. When selecting a mass spectrometer, factors such as mass accuracy, mass resolution, sensitivity, and ability to perform tandem analysis must be considered. The two commonly used ion sources are the Matrix Assisted Laser Desorption/Ionization (MALDI) source and the Electrospray Ionization (ESI) source. Each of these can ionize peptides at femtomolar levels. A MALDI source ionizes a condensed phase of a sample, while an ESI ionizes a liquid phase and thus can be coupled to in-line high performance liquid chromatography (HPLC) for sample separation. Ionization by MALDI results in singly charged ions, while ESI can assign multiple protons to the resulting ions 12. Theoretically, either source can be coupled to any analyzer and work well under optimal conditions.

The details of the five common types of analyzers will not be covered, but the designations and a few key details of their differences are mentioned here and an analysis of the three most frequently used are listed in Table 1. All rely on the application of an electric field, a magnetic field, or an electromagnetic field to isolate ions based on their m/z ratio and analyze the resultant ion mass 13. The five types of analyzers are: quadrupole, ion trap, time-of-flight (TOF), Fourier transform ion-cyclotron resonance (FT-ICR), and Orbitrap. Quadrupoles and TOF analyzers can only perform single MS scans, while ion traps and FT-ICR can perform single (MS) and tandem (MS/MS) analysis. An Orbitrap has a unique manner of operation involving an in-line ion trap 12. The combination of source and analyzer used depends on the nature of the samples to be analyzed and the desired data. Considerations such as time, cost, and instrument output must be made for optimal mass spectrometer selection.

The two most common proteomic analyses performed via a mass spectrometer are either traditional “shotgun” proteomics, known as data-dependent acquisition (DDA), or more specific data-independent acquisition (DIA). In DDA-based proteomics, protein samples are digested into peptides and injected onto the instrument to be ionized and analyzed 14. The most abundant peptides in the initial MS scan of each injection are isolated for further fragmentation, and these ions are detected and reported 14. This approach is particularly effective for conducting knowledge-blind MS analysis of samples to identify differentially expressed peptides between diseased and healthy samples that may illuminate potential biomarkers of that disease 15. Because of this capacity for broad identification, DDA comprises the “discovery” portion of the biomarker pipeline, and it is described as such in the remainder of this review article. Two combinations of ion sources and mass analyzers are common for discovery, MALDI-TOF MS and LC-ESI-MS/MS, where the tandem MS source is likely to be a combination of analyzers mentioned previously 16.

While DDA enables the discovery of potential biomarkers, DIA is a popular approach for developing targeted assays that enable more specific and reproducible results 14. DIA allows scientists to more sensitively and specifically quantify peptides of interest with high accuracy through the development of targeted assays that define a narrow m/z window to analyze a peptide of known mass 15. This process yields higher confidence about how a protein of interest in a sample is regulated between treated and untreated samples of disease 17-19. These targeted approaches are more effective at validating an observed effect than identifying one, and thus they are more commonly used for the validation and verification steps of biomarker identification, rather than discovery, and we describe them as such in this review article. DIA is commonly performed using either selected-reaction monitoring (SRM) or parallel-reaction monitoring (PRM). Frequently, targeted proteomics requires the in-series combination of multiple mass analyzers, with a triple quadrupole serving as the optimal mass spectrometer for SRM or PRM 20, 21. SRM and PRM both involve the identification of a known ionized peptide fragment, or ion, through specification of its precursor mass and application of a narrow m/z peptide selection window 22. In SRM, only the third quadrupole is involved in highly accurate and sensitive analysis of these peptide fragments of interest, whereas in PRM all three quadrupoles use high resolution for mass analysis 22. DIA typically presents a more sensitive and reproducible mass spectrum analysis, thus making it ideal for validating differential expression of proteins across samples. Additionally, relative quantification is easy to perform with DIA, allowing scientists to reveal more information across samples with each successive run 23. A third form of DIA, known as sequential window acquisition of all theoretical mass spectra (SWATH-MS), has emerged as an additional method for identifying biomarkers. Unlike SRM and PRM, in which targeted peptides need to be determined prior to data acquisition, SWATH-MS is an “untargeted” method of DIA that measures all intact precursor ions 24. For our review we have highlighted discoveries using targeted DIA; SWATH is detailed elsewhere 25-28.

Disease control and therapeutic development through biomarker exploitation is an involved process requiring three checkpoints: discovery, validation, and verification. A mass spectrometer can be used directly as a theranostic tool in each of these steps, reducing the overall time from biomarker discovery to drug development while increasing sensitivity and efficacy. The use of proteomics and the growing use of metabolomics in immunotherapy enable targeted immunotherapy enhancement and have led to numerous biomarker discoveries and diagnostic assays for evaluating therapeutic efficacy 29. In this review, we discuss some of the developments in immunotherapies that have resulted from proteomic workflows, highlight progress in the growing field of metabolomics, and emphasize the role of various types of mass spectrometers as theranostic tools performing the analyses necessary for improvement.

## Developments in Proteomics Workflows for Evaluating Immune Response

The immune system consists of suspension cells that are transported by the bloodstream, interacting with various tissues throughout the body. There are multiple cell types comprising the immune system, and it can be challenging to isolate the cell type of interest 30. Additionally, because the blood plasma that comprises these immune cells contains many highly abundant proteins compared with other tissues, the proteome of the less abundant proteins can be difficult to characterize. Methods of removing the highly abundant proteins and enriching samples for the less-abundant proteins have been described by Keshishian et al. 31. Highly abundant proteins are typically removed through a process of immunoaffinity, and the remaining sample is fractionated to reduce complexity before injection onto the mass spectrometer. This process increases sensitivity of the instrument to enhance the detection and characterization of lower-abundance proteins.

**Figure 3:**
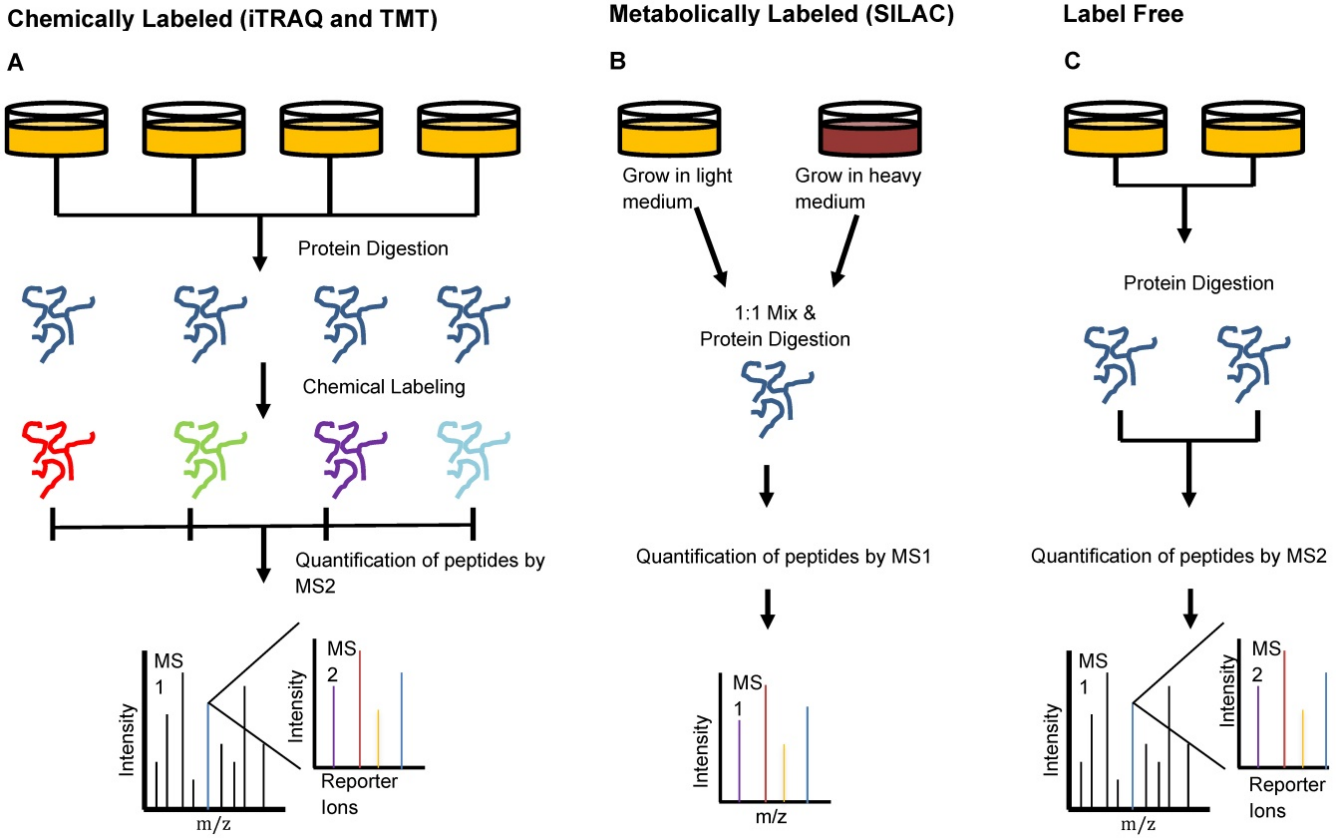
** Peptide Labeling Methods in Proteomics.** Sample preparation for injection onto the mass spectrometer can be done in multiple different manners to provide optimal quantitation. **A)** Chemical labeling can be done using iTRAQ- or TMT- isotope labels that bind to samples individually and are identified by small mass shifts during MS2. This type of labeling allows relative quantification and multiplexing of samples to enhance the high-throughput capacities of the mass spectrometer. **B)** Metabolic labeling involves the use of isotope-labeled amino acids in cell media to promote incorporation of isotopically-labeled amino acids in proteins formed by the cells during growth. This method also enables multiplexing of samples and subsequent identification of each using MS1 analysis. **C)** Label-free quantitation involves MS analysis without the addition of any label but cannot be used when interested in relative quantification or multiplexing.

Keshishian et al. combined abundant protein depletion with peptide-level fractionation to analyze 16 plasma samples from patients with acute myocardial injury. The group used immunoaffinity depletion to remove 14 of the most abundant proteins 31. They performed analysis using both isobaric mass tag labeled and label-free peptides, two of three common MS peptide labeling techniques which we have depicted in Figure 3. Isobaric mass tag labeling is an important technique used in proteomics to enable multiplexed sample analysis for high throughput relative quantification 32. This technique involves the addition of isotopic variants of a chemical marker from a set to multiple samples, which are then pooled and analyzed together by MS. Multiple forms of isobaric mass tags exist that can be selected based on the number of samples that are desired for multiplexing and the chemical structure of the peptides of interest 32. Two common commercially available mass tag labels are the tandem mass tags (TMT) and the isobaric tags for relative and absolute quantification (iTRAQ). From iTRAQ-labeled samples, the group identified 4600 proteins with a peptide false discovery rate (FDR) of <1.5% 31. This level of detection through chemically-labeled samples enabled the identification of more than 300 novel candidate biomarkers of acute myocardial injury, a number six times greater than that which was observed in the label-free study 31.

Immune cells must be able to distinguish diseased cells from healthy cells for the immune system to be effective. As previously described, the method of interaction between immune cells and host cells relies on the presentation of peptides on the cell surface of APCs via MHCs 9. APCs sample both endogenous and exogenous proteins and present peptide antigens that are generated through proteolytic excision of these proteins. Because these peptide antigens bind to MHCs on the cell surface, many of them have highly conserved regions known as anchor residues 9. When studying these peptide antigens by proteomics, it is important to have methods for extracting the antigenic material from the cell surface without disrupting their structure. Purcell et al. have compiled a review detailing multiple methods of sample processing that enable extraction of intact peptide antigens for MS analysis 33.

The first of these approaches involves a general extraction of peptides from whole-cell lysates. The cells are treated with an aqueous acidic solution and then lysed. These samples can be fractionated to reduce overall complexity and enhance instrument sensitivity and run directly on a mass spectrometer via either DDA or DIA. The method is relatively straightforward, but can result in samples that are saturated with highly abundant proteins due to the lack of filtration or depletion 33. This can diminish the detection of less abundant proteins that may convey more information about the biological processes of interest, thus reducing MS sensitivity relative to other sample preparation protocols 33. The second method described is a non-lytic approach where cell surface-bound peptides are dissociated from the cell surface by a wash with an isotonic buffer. This approach has the advantages of not affecting cell viability and of being more sensitive than the whole-cell lysate method, but still often requires the aid of biological assays to identify which peptides are most interesting for biochemical characterization 33. The final method described by Purcell et al. involves the use of monoclonal antibodies in immunoaffinity chromatography to enrich samples for MHC-binding peptides by isolating highly specific peptides expressing anchor residues. Peptides extracted from samples using any of these methods can be subsequently identified using mass spectrometry.

**Figure 4:**
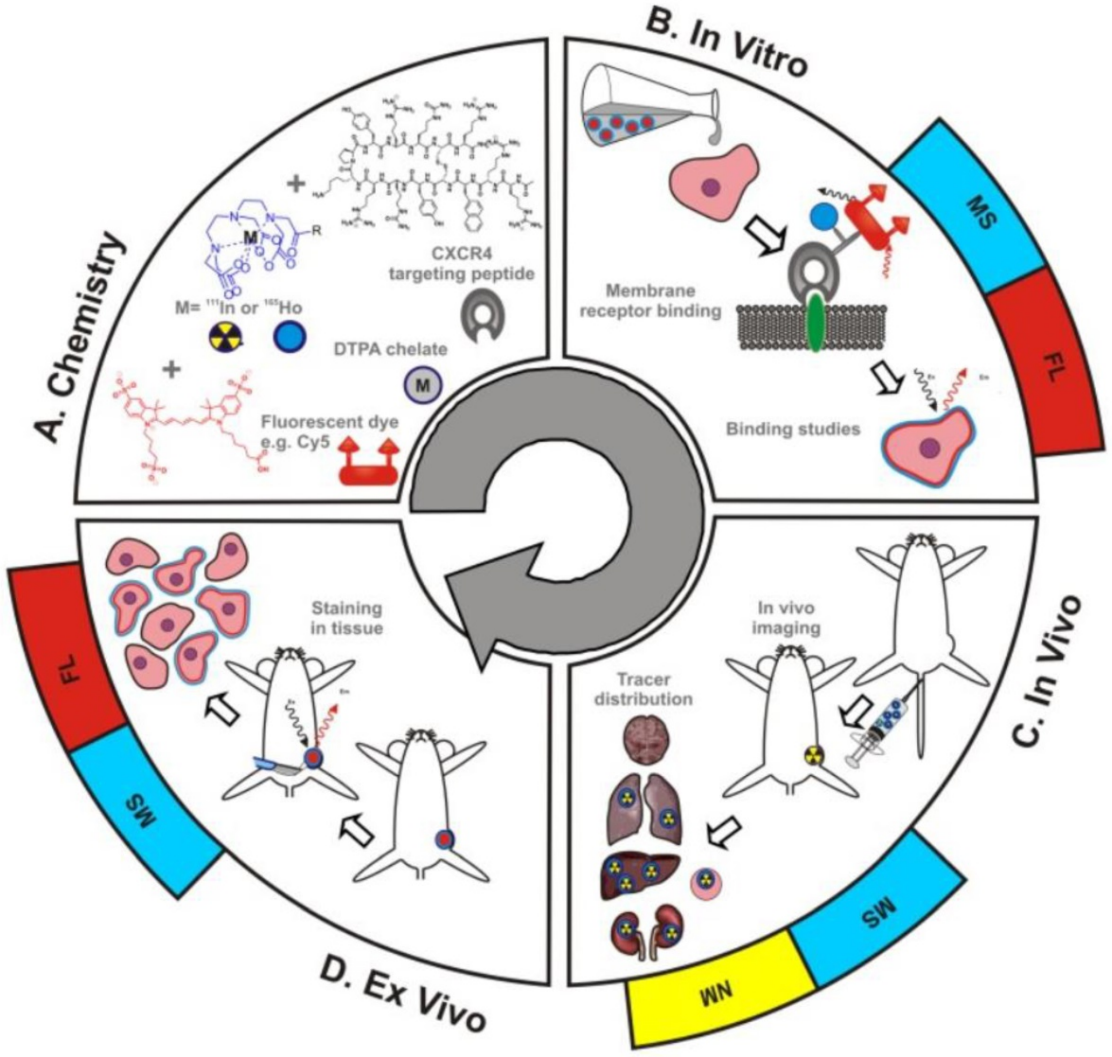
** Schematic overview of the use of hybrid tracers in theranostic applications A)** Components that make up the hybrid tracer used to target CXCR4: a Cy5-fluorescent dye, a DTPA-chelate and the CXCR4 targeting peptide Ac-TZ14011. After functionalization with either a radioisotope (radiolabel; yellow) or a non-ionizing lanthanide isotope (blue), this tracer also becomes of value for respectively nuclear medicine (NM) or mass spectrometry (MS) based applications. **B)** *In vitro* this tracer can be used in fluorescence (FL)- (red) and MS-based cytometry and imaging studies. **C)** *In vivo* NM-based imaging studies can be complemented with NM- or MS-based analysis of uptake levels in tissues and **D)** ex vivo FL- and MS-based imaging could be used to evaluate the degree and heterogeneity of tissue staining following *in vivo* tracer administration 23.

MS integration into theranostic applications has been limited by an inability to directly compare MS with the traditional theranostic methods of fluorescent imaging and radioactivity. As shown in Figure 4, Buckle et al. have recently developed a hybrid tracer that allows fluorescent imaging, MS, and nuclear medicine to be utilized simultaneously 34. The hybrid tracer is composed of Cy5-fluorescent dye, a DTPA-chelate, a CXCR4 targeting peptide, and a non-ionizing lanthanide isotope or radioisotope. CXCR4, or chemokine receptor 4, is an oncologic target that is involved in the primary growth, angiogenesis, tumor invasiveness, and metastasis of cancer 35. Meta-analysis of breast cancer patients proved that overexpression of CXCR4 was significantly associated with poor prognosis 36. The targeting peptide for this study was Ac-TZ14011, as it has been proven to successfully home to CXCR4 37,38. A single DTPA-chelate functionalized Ac-TZ14011 for MS detection, and the addition of Cy5-fluorescent dye and a radioactive isotope allowed for fluorescent and radioactivity analysis respectively 34. The MS analysis was compared with fluorescence or radioactivity at all phases of drug characterization -*in vitro* , *in vivo* , and ex vivo. The *in vitro* receptor affinity of the targeting peptide was determined by inductively coupled plasma mass spectrometry (ICP-MS) and fluorescence-based flow cytometry. Laser ablation ICP-MS (LA-ICP-MS) imaging and fluorescence-based imaging evaluated the microscopic binding pattern of the tracer in tumor cells, ICP-MS and radio-detection imaging assessed *in vivo* biodistribution patterns, and LA-ICP-MS imaging and fluorescence-based imaging investigated the molecular pathology of excised (ex vivo) tissue samples. The MS data was homogeneous to the fluorescence and nuclear medicine data, indicating that adopting a hybrid tracer allows for MS-based analysis in conjunction with fluorescent and nuclear medicine methods.

## Data-dependent Acquisition and Biomarker Discovery

Mass spectrometers have the unique ability to study host-pathogen and normal-malignant interactions *ex vivo* due to their potential for label-free detection and identification of molecules directly from tissue samples 39, 40. Based on statistical analysis, MS can efficiently identify biomolecules that are upregulated in pathogenesis and further characterization of these molecules can confirm their involvement in disease 41, 42. Many immunotherapies are designed to target cancer cells and have been successful in treating non-solid blood cancers but have had variable success with solid tumor treatment, highlighting the need for improved targets to broaden the spectrum of cancers ameliorated by immunotherapies 43-46. As discussed in this section, MS has been used to discover biomarkers in cancers, infectious diseases and autoimmune diseases, and has recently been paired with next generation sequencing to study the immune repertoire.

### Cancer neoantigens

Mass spectrometry can aid in the discovery of neoantigens, which can be used to create clinically successful personalized cancer vaccines 47-49. Tumors experience a striking number of somatic mutations, and this can result in epitopes derived from neoantigens presented on the cell surface via MHC molecules 50, also known in humans as human leukocyte antigen (HLA) complexes. For over two decades MS has enabled identification of tumor associated antigens 51, and developments in sensitivity and specificity have allowed MS to be instrumental in the more recent discover of neoantigens.

Neoantigens of murine and human origin, discovered by MS and immunoassays, confirm the immunogenicity of the targets 52, 53. The neoantigens are discovered by first isolating HLA complexes from cancer cells, and then using LC MS/MS analysis to analyze the resulting displayed peptides. The peptides are then identified by comparing the MS scans to reference databases. T-cell assays are used to validate the selected neoantigens as capable of inducing an immune response. The immunogenic neoantigens can subsequently become the target of various immunotherapies, such as cancer vaccines and TCR-engineered T cell therapy 54, and further used to monitor the efficacy of the immunotherapies. Bassani-Sternberg et al. employed MS on native human melanoma tissue and discovered multiple clinically relevant neoantigens 55. Over 95,500 melanoma-associated HLA isolated peptides were processed by MS, and ultimately eleven mutated peptide ligands were selected for further analysis as they were present on tumor tissue samples carrying somatic mutations. Neoantigen-specific T cell responses confirmed the immunogenicity of four of the eleven selected peptide ligands, validating the efficacy of the MS analysis. In a parallel study, myeloma-associated T cell antigens on the HLA ligandome level were characterized by MS, resulting in fifty-eight highly specific antigens 56. The target antigens were subject to preexisting T cell responses in multiple myeloma (MM) patients, which implements the antigens in the pathogenesis of MM. *In vitro* assays proved that the antigens evoke peptide-specific T cell targeting in response-naïve MM patients, highlighting the potential use of the discovered neoantigens for T cell-based immunotherapy of MM. This rapid, accurate discovery of neoantigens enables the development of personalized cancer vaccines and T cell therapies.

### Cancer Biomarkers in Peripheral Blood Mononuclear Cells

LC MS/MS analysis was used to identify candidate biomarkers in the peripheral blood mononuclear cells (PBMCs) of pancreatic cancer patients. This study compared PBMCs of three groups of patients to infer biomarkers from differentially expressed proteins among the PBMCs: healthy individuals, individuals with benign diseases, and individuals with pancreatic cancer. Li et al. prepared the PBMCs using whole cell lysate and digestion protocols and analyzed the samples by conventional LC MS/MS instrumentation. A fold-change (FC) threshold of FC < 0.8 or FC > 1.2, as well as a p-value of <0.05 and an FDR <1% was used to determine whether a protein was differentially expressed across samples. From this analysis, 3357 proteins were identified, 114 of which were differentially expressed in the pancreatic cancer patients compared to expression in either the healthy patients or in those with benign disease. Of those 114 differentially expressed proteins, 35 were upregulated in the pancreatic cancer patients and 79 were downregulated. The differentially expressed proteins were grouped based on physiological functionality to gain insight into pathway enrichment in the presence of pancreatic cancer. The top five enriched categories included hematological system development and function, immune cell trafficking, lymphoid tissue structure and development, connective tissue development and function, and organismal development. These differentially expressed proteins present 114 novel potential candidates for biomarkers that are indicative of pancreatic cancer. From various epitopes of these biomarkers, immunotherapies could be designed, enhancing treatment of pancreatic cancer and enabling earlier detection of the disease 57.

### Infectious Diseases

MS has been instrumental in finding new information about cancer and is experiencing the same success in identifying influential biomarkers of infectious diseases. Interest in understanding the impact of bacterial infection through MS analysis stretches back to 1998, when Uttenweiler-Joseph et al. used MALDI-TOF MS to analyze differentially expressed peptides induced during an immune response of bacterial-infected *Drosophila*. Despite the low number of *Drosophila* peptides that had been identified and characterized at the time of publication, this group was able to detect 24 peptides in the hemolymph of the flies induced by immune response both 6 hours after infection and 24 hours after infection by bacteria. Of these 24 differentially expressed peptides, the group was only able to identify 4 that had been already characterized and began to speculate on their involvement in the immune response. Following the four identified peptides across a period of three weeks, this group was able to detect levels of two of the differentially upregulated peptides for two weeks after infection, likely making these peptides prime biomarkers for infection by this bacterium in *Drosophila* 58*.* Though this experimentation was ahead of its time and had a lack of available reference information to conduct a thorough MS study, the Uttenweiler-Joseph et al. publication illustrates the extreme growth that this field has seen since conception, as since 1998 MS has experienced a transformation in its ability to identify disease related biomolecules. More recent MS experiments involving analysis of infectious disease have yielded more fruitful results, thanks to the compounding libraries for identifying the biological role of molecules. Two such cases, investigating *Salmonella* and *Francisella*, involve the discovery of critical metabolites and are discussed in the section on metabolomics.

### Autoimmune Disease

MS reaches beyond the implications of cancer and infectious diseases and can allow us to elucidate biomarkers of autoimmune disorders, such as rheumatoid arthritis (RA). Estelius et al. were interested in characterizing patient response, in terms of protein regulation, to the treatment of RA by an antibody, infliximab, that blocks tumor necrosis factor (TNF). To perform this analysis, lumbar puncture derived cerebrospinal fluid (CSF) samples of 10 patients suffering from RA were obtained before and after treatment with the drug. Samples were subjected to proteomics workflows and analyzed using mass spectrometry based quantitative proteomics. The group found that 35 proteins exhibited decreased expression in the patients following treatment by infliximab and found that many of these differentially regulated proteins were predominantly involved in inflammatory processes of the immune response. Since RA is often characterized by pain that is caused by inflammation, this drug works to diminish the inflammatory response, thereby reducing associated pain 59. This study is important for illuminating the systemic impacts that a drug reducing the inflammatory response can have, and for determining if drugs such as these will have unintended impacts.

### MS and Next Generation Sequencing for Studying the Immune Repertoire

MS has been paired with next generation sequencing (NGS) to obtain a more comprehensive understanding of the immune repertoire, which are the adaptive components of the immune system, produced by B and T cells. B cell immunoglobulins (antibodies) undergo recombination of the variable, diversity, and joining gene segments to create a vast number of unique receptors for targeting foreign antigens that may enter the body, forming the subcategory of the immune repertoire known as the B cell repertoire. Recently, the characterization of B cell receptor sequencing from the peripheral blood of individuals before and after kidney transplant resulted in the detection of a common pool of immunogenic antigens that are likely to cause post-transplant rejection - information that can have clinical implications for the management of kidney transplant rejection 60. VanDuijn et al. employed NGS in tandem with MS-based proteomics to characterize the B cell immune repertoires in groups of rats after immunization with purified antigens, and demonstrated that NGS and MS-based proteomics contribute complementary as well as independent information about the repertoire 61. It is expected that NGS and MS can be used simultaneously to provide complementary insight into B cell repertoires.

We have demonstrated the importance of mass spectrometers in the discovery and identification of potential novel biomarker candidates across cancers, infectious diseases, and autoimmune disorders. These instruments have the capacity to not only discover biomarkers of disease, but also to validate their exact role in disease progression or remission. This information can help lead to the development of immunotherapies targeting biomarkers or tools for monitoring these markers as signs of successful treatment, thus illustrating the essential role of proteomics in both the development of immunotherapeutics and in the validation of therapeutic efficacy.

## Targeted Data-independent Acquisition (DIA) for Biomarker Validation and Verification

DDA-based MS is particularly useful for discovering biomarkers implicated in diseased cells, but more work must to be done to reveal the role that these biomarkers play and to what extent they can be exploited for therapeutic development and in the validation of therapeutic efficacy. DIA-based MS offers an optimal platform to explore the role of these biomarkers, to enable their relative quantification and to validate that they are essential markers of disease or of disease remission 20,21. Here we present the research of multiple groups that have developed MS pipelines using DDA-based MS to identify biomarkers and then subsequently using DIA approaches to validate their findings. The validation and verification of biomarkers results in novel, definitive targets for diagnostic testing as well as immunotherapies.

### Cardiac Injury

Addona et al. capitalized on the high-throughput capacity of MS to identify candidate biomarkers of cardiac injury without the use of antibody-based assays requiring multiple general antibodies. To perform this analysis, the group sampled blood directly from patients' hearts before, 10 minutes after, and 60 minutes after receiving planned myocardial infarction (PMI) to treat hypertrophic cardiomyopathy to analyze for biomarkers appearing as indicators of injury. The group developed a pipeline to identify and subsequently validate the plasma protein biomarkers. Initially, a general mass spectrometer scan employing DDA was used to discover candidate biomarkers. An FC > 5 and FDR < 1.5% were required as indicators of differential expression compared to baseline by the group to enable identification of 121 proteins, 40 of which were observed across all three patients evaluated. Label-free targeted high-performance LC MS/MS accurate inclusion mass screening was used to qualify the candidates observed by DDA. Finally, verification of the discovered and qualified candidates was performed by employing a targeted, quantitative DIA MS-based method using isotope labeled peptide standards (SID (stable isotope dilution)-MRM-MS). The information identified by this study provided multiple markers of myocardial infarction that can be targeted by immunoassays as indicators of disease for thousands of potential patients 62. Further testing may reveal which protein biomarkers play active roles in causing cardiac injury, and lead to unique immunotherapies counteracting these specific proteins.

### Kidney Disease

Diabetes remains a prominent autoimmune disease worldwide and is the largest cause of kidney disease. The current gold standards for recognizing early stage diabetic kidney disease are relatively poor diagnostic tools that evaluate either the urinary albumin creatinine ratio (ACR) and/or the estimated glomerular filtration rate (eGFR). Bringans et al. hypothesized that the use of mass spectrometry-based proteomics would expand the identification of biomarkers and allow for the development of improved assays for early detection of this disease 63. Using plasma from a group of patients that were subdivided into three stages of kidney disease, normo- micro- and macro-albumeric groups, samples were prepared for DDA-based MS analysis. The samples were labeled using iTRAQ chemical labels and were analyzed via LC MS/MS.

From this DDA, 32 proteins were identified as potential biomarkers of disease. For each of the 32 identified proteins, targeted MRM assays were developed to validate the presence of these proteins in patient samples and to detect low abundances of peptides corresponding to these proteins. A larger subset of patient samples was used to validate the implication of these biomarkers in development of kidney disease. 25 proteins met the criteria set for validation by this group, and 8 of these 32 original protein biomarkers were found to be differentially expressed between patients belonging to each subset group of kidney disease. An even larger set of patient samples was used to test whether these biomarkers worked as early markers of kidney disease, and it was found that 5 of the 8 differentially expressed proteins significantly correlated with the predictions of ACR and another 5 with those of eGFR. The prediction capacity of this targeted MS model compared to the gold standard ACR and eGFR tests was encouraging, as the eGFR diagnosis model using targeted MS showed an improved True Positive rate of 88% vs 73% and a reduced False Positive rate of 32% vs 40%. The ACR diagnosis model using targeted MS showed an improved True Positive rate of 32% vs 40%, but an increased False Positive Rate of 15% vs 8%. These results showed that the eGFR diagnosis model obtained from the development of the targeted MS assay based on biomarkers identified by DDA-based MS was an improvement over the current gold-standard approaches, enabling earlier detection of kidney disease and increased potential of intervention. Identification of these biomarkers also creates the opportunity for immunotherapeutic development by providing targets for immunotherapies to recognize. This study clearly illustrates the advantages of using MS-based approaches for biomarker identification, verification, and validation 63.

### Minimal Residual Disease in Multiple Myeloma

In the presence of immunotherapies, it is sometimes difficult to distinguish markers of disease from markers of the therapeutic. This is observed in the treatment and subsequent monitoring of multiple myeloma (MM) to ensure reduction of minimal residual disease (MRD). MM is often treated through combinations of multiple antibodies, but the malignancy of B cells is monitored by the production of M-protein, a monoclonal immunoglobulin. Conventional technologies, such as flow cytometry or serum electrophoresis, are not sensitive enough to distinguish M-protein from general antibodies, or to detect low levels of M-protein that are observed during MRD. Zajec et al. developed a targeted mass spectrometry assay measured by PRM to detect M-proteins from the serum of patients and to distinguish it from therapeutic antibodies. This assay involves the incorporation of stable isotope labeled peptides as internal standards for absolute quantification to detect two common and specific peptides of M-protein. The group tested their assay on 10 patients that, by flow cytometry analysis, had no detectable disease. Of these 10 patients, all of them had detectable disease when analyzed by this targeted MS assay, rendering this assay more than two orders of magnitude more sensitive to M-protein levels than the conventional M-protein diagnostic tests. Additionally, samples for MS analysis were obtained by serum sampling rather than by bone marrow biopsy, providing a far less invasive alternative to current M protein monitoring protocols 64. This study clearly shows the advantages of MS-based technology for evaluating the efficacy of immunotherapies in terms of sensitivity, specificity, and patient sampling.

### Checkpoint Inhibitors

**Figure 5:**
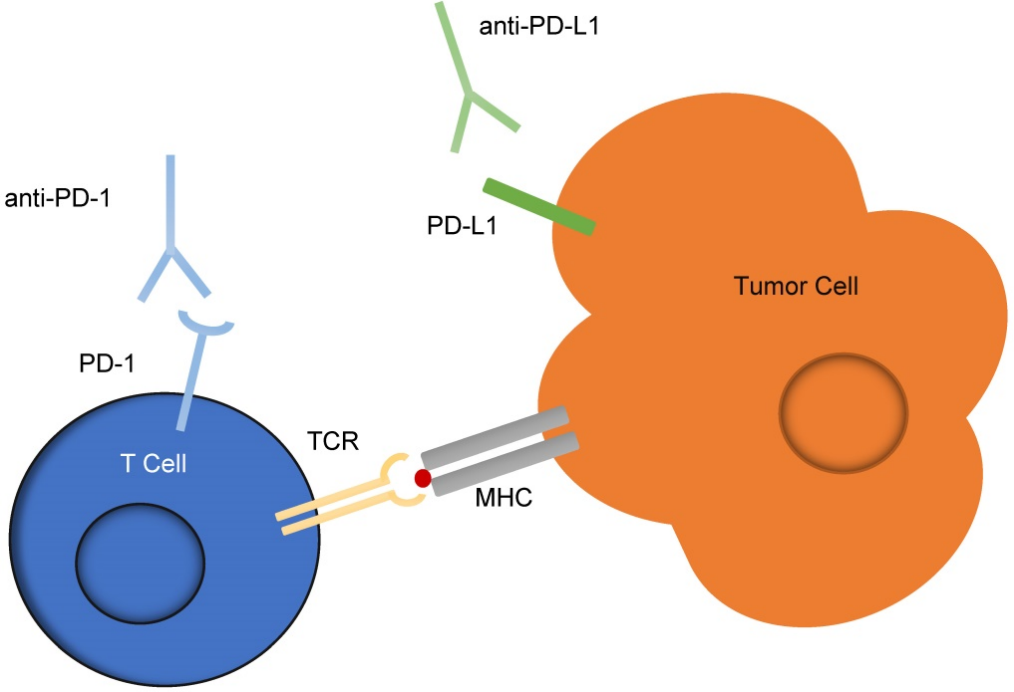
** Checkpoint inhibitors targeting the PD-1 and PD-L1 interaction between T cells and tumor cells.** An interaction between PD-L1 expressed on tumor cells and PD-1 expressed on T cells often acts as a suppressor for T-cells activated against signs of disease. This schematic illustrates the use of anti-PD-1 and anti-PD-L1 antibodies, known as checkpoint inhibitors, to reduce the potential of this suppression and enable the T-cells to act against cancerous cells.

Checkpoint inhibitors are one of the most successful forms of immunotherapy implicated in cancer treatment. Checkpoint inhibitors targeting the programmed cell death-1 (PD-1) receptor on T-cells and the programmed cell death-1 ligand 1 (PD-L1) expressed by cancer cells, shown in Figure 5, are the most common, and patient tumors are often evaluated for overexpression of the PD-L1 marker before deciding whether to employ this treatment. Currently, the test for overexpression uses immunohistochemistry (IHC) to detect levels of PD-L1. However, it has been suggested that these IHC diagnostic tests are unreliable, as half of patients indicated as having PD-L1 positive tumors by IHC do not respond to PD-L1 therapeutics, and 15% of those which are deemed negative for PD-L1 overexpression by IHC do respond to the therapeutic 65, 66. An additional marker expressed by cancer cells, programmed cell death-2 ligand 2 (PD-L2) has been shown to have a two- to six-fold higher affinity for PD-1 than does PD-L1, but because high quality antibody reagents for PD-L2 are unavailable, IHC analysis is not a good indicator of PD-L2 abundance 67.

Hypothesizing that the IHC assay is not sensitive enough to detect levels of PD-L1 and PD-L2 that might indicate the efficacy of the checkpoint inhibitors, Morales-Betanzos et al. developed targeted MS assays to enable the measurements of PD-1, PD-L1, and PD-L2 in sections of human melanoma biopsies 67. These targeted assays were performed on 22 sections of melanoma tissues from pre-treated biopsies or surgical resections from patients treated with checkpoint inhibitors to evaluate levels of PD-1, PD-L1 and PD-L2. PD-L1 abundances measured by MS were compared with IHC assessments and although a significant correlation (Spearman r = 0.5841, p = 0.0054) was observed, MS measurements enabled detection of N-glycosylated forms of the PD-L1 that were not detectable via IHC. Additionally, targeted MS measurements enabled detection of PD-L2, revealing that PD-L2 expression in melanomas is comparable to PD-L1 expression. IHC analysis is unable to detect PD-L2 or post-translationally modified forms of PD-L1 at this time, and these limitations of IHC analysis are overcome by MS technology. The new information that is made possible by MS illuminates a possible explanation of why sometimes the checkpoint inhibitors work when IHC analysis predicts that they will not. From this study, it was shown that IHC is inadequate at predicting drug efficacy while the targeted MS-based approaches maintain the currently observed specificity but also represent a dramatically improved sensitivity to PD-L2 and modified PD-L1 expression 67. This targeted MS assay presents a much better predictor of drug efficacy based on PD-L1 and PD-L2 expression while also illuminating other potential biomarkers, again clearly showing the advantage of MS-based approaches for evaluating and improving immunotherapies.

### Graft-versus-Host-Disease

One of the major drawbacks associated with adoptive T cell therapy is the development of graft-versus-host disease (GVHD) when introducing T cells from one patient into another patient. The root cause of this reaction is a mismatch in minor histocompatibility antigens (mHAs) from the host, usually in the form of single-nucleotide polymorphisms (SNPs). These antigens form a specific lock-and-key like bond with T-cell receptors, and when a T-cell recognizes a mismatch it can trigger the immune response GVHD. In the case of T-cell receptor (TCR) and chimeric antigen receptor (CAR) engineering, the objective is to mimic this type of response against leukemia cells, and the effect is known as graft-versus-leukemia (GVL). Lansford et al. were interested in using MS data to develop a model that would predict general, publicly usable mHAs with high-binding affinity to HLA in multiple patients based on analysis of their genome to promote GVL, reduce GVHD, and enhance immunotherapies against leukemia 9.

Lansford et al. sampled 101 patients diseased with either acute myeloid leukemia (AML), chronic myeloid leukemia (CML), myelodysplastic syndrome (MDS), or myeloproliferative neoplasms (MPN) both before and after reception of grafts from either HLA-matched donors or unmatched donors. These samples were subjected to both genomic and MS analysis to build a model to identify leukemia-associated mHAs with desirable properties for public use. The desirable properties consisted of: presentation on a common HLA, high binding affinity, expression in AML but not in GVHD target organs, and optimal allele frequency to allow minor mismatches. Using this approach, the model predicted 102 novel public GVL mHAs that fit the criteria. Lansford et al. then validated the model using targeted MS coupled to differential ion mobility spectrometry (DIMS-MS) to filter out competing peptides and cleanly confirm expression of target mHAs based upon two complementary features: concordance of the fixed compensation field (Ec) of maximal transmission between the model peptide and the sample, and a direct comparison between the model peptide and the sample MS/MS. Using DIMS-MS, they were able to confirm one predicted GVL mHA as a potentially useful immunotherapy target 9. This highly innovative combination of computational modeling, MS and genomics yields a potentially major innovation for reducing the potential of GVHD and enhancing the effect of GVL, reducing the toxicity and enhancing the efficacy of immunotherapies.

Discovery, validation, and verification remain the three important stages in the pipeline of biomarker discovery by MS-based proteomics, illuminating peptides specific to disease and indicative of immune response that can help guide our development of therapeutics and evaluate the efficacy of treatments.

## Metabolomics

**Figure 6:**
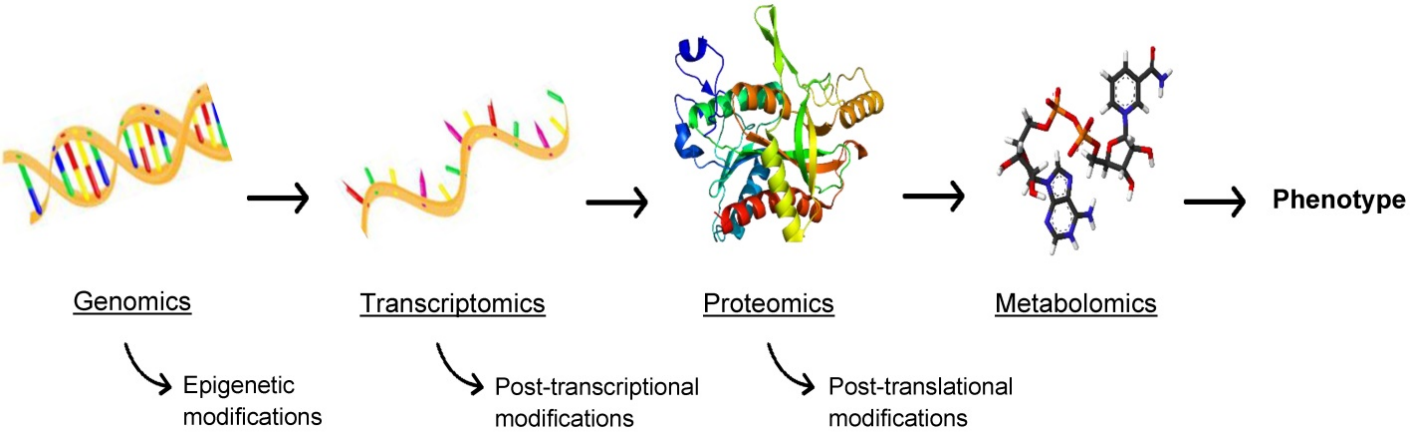
** The -omics cascade.** The -omics cascade follows the journey from genotype to phenotype. While genomics, transcriptomics, and proteomics are essential to understanding disease, metabolomics is more closely linked to phenotype and metabolites are not readily subject to ex post facto modifications.

While this review is primarily focused on proteomics for theranostic enhancement of immunotherapies, it is necessary to discuss the burgeoning role of metabolomics, the comprehensive analysis of metabolites in biological samples. Metabolism is at the cornerstone of life, the biochemical energy transfer that dictates the growth, differentiation, and survival of cells in both a healthy and diseased state. Metabolites (e.g. ATP, acetyl-CoA, NAD+), are functional readouts of a cellular state and therefore more closely linked to phenotype than genes and proteins, as depicted in Figure 6, which are subject to epigenetic regulation and post-translational modification 68. As a diverse population, with varying chemical properties and abundance, metabolites present a challenge for traditional MS, but methods have been developed to assess the metabolome using MS and MSI 69. Similar to proteomics, metabolomics involves analysis through both targeted and untargeted assays coupled to computational tools and databases. A broad range of instrumentation can be used to perform metabolomics, such as equipment specific to the isolation of redox active compounds (coulometric array detectors), lipids (evaporative light-scattering detectors), and metals (inductively coupled mass spectrometers) 70. The three central technologies that have emerged as the drivers of metabolomics are NMR spectroscopy, LC-MS, and GC-MS 70. These methods allow high throughput analysis of a multitude of small molecules 71, allowing for the expansion of the metabolomics library. In consonance with peptide expression as an indicator of disease, the abundance of certain metabolites can signify a disease state, allowing for improved diagnostics and disease monitoring. Metabolites also interact with and can be the target of therapeutics, making metabolomics a valuable field for advancements in immunotherapy.

### Cancer and Cardiovascular Disease Diagnosis

Despite recent advancements, cardiovascular disease and cancer remain the two leading causes of death in the United States 72. Metabolomics has already shown promise as a theranostic tool for the enhancement of immunotherapies against cancer and cardiovascular disease. Cancerous tumorigenesis relies on the reprogramming of cellular metabolism, as cancer cells must extract nutrients from an often nutrient-deprived environment to grow 73, and through metabolomics we are illuminating metabolites essential to cancer progression and immunosuppression. A major roadblock limiting the success of immunotherapies against solid tumors is the solid tumor microenvironment (TME), and the balance of glucose, glutamine, asparagine, and other metabolites can create a TME that favors cancer cells and regulatory immune cells while inhibiting antitumor immunity 73. The efficient and accurate monitoring offered by metabolomics can help us better prepare immunotherapies for enhanced solid tumor efficacy by providing a comprehensive understanding of metabolite expression in the tumor microenvironment that immunotherapies must overcome for functionality. Additionally, MS technologies allow detection of the subtle changes in metabolites that can provide insight into biological pathways and an improved understanding of cancer 74. Metabolomic workflows that enable detection of biomarkers of disease and disease microenvironments can be applied to many diseases involving transport of metabolites, allowing for the enhancement of diagnostic tools and immunotherapeutics and making metabolomics an indispensable theranostic tool of immunotherapy.

Two examples of the promising clinical applications of metabolomics are using urine for cancer diagnosis 75 and identifying cardiovascular disease biomarkers 76. Urine monitoring has long been used for diagnostics and is now being analyzed by mass spectrometry for the detection of cancer in organs directly associated with urine (bladder, prostate, kidney cancer) as well as remote cancers such as breast, pancreatic, and lung cancer. Metabolomic study of urine is challenged by sample complexity, low concentrations of metabolites, and a lack of standards to quantify the findings. Recent advances in MS and urine processing, such as the use of inductively coupled plasma (ICP) MS, and supercritical fluid chromatography respectively, have mitigated some of the challenges, allowing for the identification of cancer biomarkers and indicating metabolic pathways favored during malignancy 75. A targeted study employing LC-MS and GC-MS enabled the identification of nucleosides 1-methyl-adenosine and 3-methyluridine as proposed biomarkers for ovarian cancer 77. Further evaluation of these markers illuminated that cancer pathogenesis is closely linked to DNA methylation processes 77. In breast cancer patients, succinate, a metabolite of the TCA cycle, was shown to be present at significantly higher levels as compared to healthy controls 78. Using urine as a diagnostic has several benefits (noninvasive, relatively high quantity can be provided), and the information gathered using MS reveals which cancer associated metabolic pathways should be targeted by immunotherapies. New biomarkers can provide insight into disease progression which can in turn improve the design of immunotherapies, and monitoring changes in the presence of biomarkers can indicate therapeutic efficacy. In combatting cardiovascular disease, large strides have been made in understanding heart failure (HF) using metabolomics 76. One such finding, after investigation of mice and human HFs using LC-MS/MS and GC-MS metabolomics paired with transcriptomics, discovered that the branched chain amino acids (BCAA) catabolic pathway is downregulated during heart failures, generating an accumulation of BCAA 79. Subsequent studies proved that pharmacological enhancement of BCAA catabolism delayed heart failure progression in a mice preclinical model 80. By using BCAA and other metabolites identified by metabolomics methods, novel immunotherapies for cardiovascular disease can be formulated.

### Macrophages and Fibroblasts in the Tumor Microenvironment

As many developments share the goal of eliminating cancer, methods of analyzing immune cells specifically by mass spectrometry are essential. A growing body of work supports the notion that the tumor microenvironment, which is comprised of all the non-cancerous cells that surround and interact with the tumor cells, is closely connected to each stage of tumorigenesis, including initiation to progression to metastasis 81, 82. A few of the cellular populations that contribute to the immunosuppressive tumor microenvironment include cancer associated fibroblasts, myeloid derived suppressor cells, T regulatory cells, and tumor associated macrophages 83. These circulating cells are accompanied by physical alterations such as abnormal pH and low oxygen concentrations 84. The rapid advancements in nanotechnology have paved the way for nanotheranostics to target and perform diagnostics of the tumor microenvironment, as shown by nanoprobes that respond to hypoxic conditions or the overexpression of antigens on macrophages 85. As we gain a deeper understanding of the biomarkers and biomolecules that indicate a switch from a homeostatic to pro-tumorigenic state through the application of MS, TME-specific nanotheranostics and immunotherapies will be more effective in cancer diagnosis and therapy as strictly defined phenotypes are targeted. For example, Ouedraogo et al. have developed an efficient MALDI-based method for examining eukaryotic cells and applied the method to studying polarization phenotypes of macrophages 86. MALDI-TOF MS has been used extensively to identify molecules and biomarkers and has recently been used for whole cell analysis. M1 phenotype macrophages can aid in anti-tumor immunity but are converted to M2 phenotype immunoregulatory macrophages in the presence of certain cytokines such as interleukin (IL)-4, IL-10, and transforming growth factor beta 87, 88. MALDI-TOF MS elucidates the subtle changes experienced by macrophages during malignancy and can be used to develop both diagnostic and therapeutic probes targeting M2 macrophages. Macrophages are only one of the components contributing to the immunosuppressive TME, and continued work using MS has the potential to fully unravel the complex cellular puzzle that maintains the TME. This past year has already brought the groundbreaking discovery through MS proteomics that nicotinamide N-methyltransferase (NNMT) is the master metabolic regulator of cancer-associated fibroblasts (CAFs) 89, which are the dominant cellular component in the TME and perform metabolic and immune reprogramming of the TME, as well as alter the ECM structure through the release of various factors 90, 91. NNMT inhibition caused a reversion of the CAF phenotype 89, illustrating the clinical potential of NNMT as a diagnostic marker and therapeutic target. While cancer associated fibroblasts have been targeted by fibroblast activation protein (FAP), it has been shown that the enzymatic inhibition of FAP does not always reduce CAFs' role in cancer progression 92. Thus, although FAP is a promising theranostic target, there may be other biomolecules on CAFs that play a larger role in tumorigenesis. Using MS, NNMT has been identified as such a molecule 89, and further testing may reveal that NNMT is a superior target to FAP. FAP has been targeted by antibodies and CAR T cells 92, two widely adopted forms of immunotherapy, and such treatments geared towards NNMT could enhance the efficacy of immunotherapies combatting CAFs.

### Microbiome, Metabolism, and Cancer Immunotherapy

In conjunction with a rising interest in the microbiome and its role in disease and therapies, MS-based metabolomics has shed light on the interactions between the microbiome, metabolism, and cancer immunotherapy. It has been observed that bacteria use metabolites to create a biofilm which can in turn promote cancer progression 93. Four MS-based metabolomic platforms were used to assess colon cancer biofilm metabolism, and revealed that polyamine metabolites were present at a significantly higher concentration in resected cancer tissue than normal tissue, implicating polyamine metabolites in colon cancer development 93. Metabolomics can also determine the direct effect of the microbiome on the efficacy of immunotherapies. Routy et al. and Gopalakrishnan et al. observed a significant decrease in the antitumor effects of anti-PD-1 blockade due to abnormalities in the gut microbiome 94, 95, and it is likely that the influence of the microbiome extends to many types of disease.

### Infectious Disease

*Salmonella*, a gram-negative facultative anaerobe, causes almost one billion cases worldwide of foodborne illness per year 96,97. Researchers have recently applied MALDI-MSI to a *Salmonella* Typhimurium murine model of infection and discovered molecules differentially expressed during infection, one of which was palmitoylcarnitine (PalC) 98. PalC accumulated at the loci of *S.* Typhimurium infection and resulted in the disruption of mesenteric lymph nodes (MLNs), as shown by the absence of B and T lymphocytes and the altering of subcapsular sinus macrophages. Subsequent experimentation revealed that PalC significantly modulates the immune activity in MLNs. Although PalC was previously implicated in bacterial and inflammatory diseases, it was only through MSI that a direct influence on the immune response was uncovered 98. This method can be applied to other host-pathogen interactions and unveil additional compounds that can be utilized in immunotherapy.

MALDI-TOF MS allows the study of structural modifications directly from intact bacteria, without purification or chemical treatment steps. Bacteria have evolved several methods of evading host innate immunity, and a key strategy used by gram-negative bacteria is the modification of lipid A on lipopolysaccharides (LPS) 99. *Francisella tularensis*, a human pathogen that colonizes phagocytic cells, employs this evasion technique and remains hidden from Toll-like receptor 4 and cationic antimicrobial peptides 100. Using the surrogate strain *Francisella novicida*, Robert et al. cultured the bacteria in various conditions that mimic the unfavorable microenvironment within macrophages and analyzed the lipid A structure by MALDI-TOF MS. Testing of cultures with acidic pH, oxidative stress, or high concentration of divalent cations revealed that low pH results in a conformational change in lipid A in *F. novicida*. Lipid A purification and characterization can be tedious and time-consuming, whereas MALDI-TOF MS allowed for the efficient screening of over sixty conditions. Once again, the accuracy, precision, and mechanism of MS provided a means for obtaining novel insights into the pathogenicity of an organism, which can in turn lead to better diagnostic and therapeutic strategies. The characterization of LPS using MS to understand the endotoxin's molecular pathogenesis has recently been reviewed by Khan et al., and as the review discusses the MS data, it sheds light on LPS immune response, tolerance, and immune dysfunction 101.

We have shown that metabolomics is poised to play a large role in the discovery of biomarkers, molecular pathways that lead to pathogenesis, and the effects of the microbiome. The diagnostic capabilities of metabolomics are highly promising, as it is the closest member of the omics family to phenotype. Despite this, many of the causal links between metabolic biomarkers and disease remain to be verified. This issue is especially relevant to the study of the microbiome due to the complexity of the relationship between healthy cells, foreign cells, and diseased cells.

## Conclusions and Perspectives

One of the hallmarks of immunotherapy is specificity. Although recent advances allow for targeted radiation and chemotherapy, immunotherapy often finds its basis in antigen specific delivery. Despite the immune system's capacity for recognizing millions of diverse antigens, disease is still prevalent. Cancer and infectious diseases have developed mechanisms to evade the immune system, and autoimmune disorders are a result of a defective immune system. Immunotherapy works to manipulate the immune system so that it can effectively fight disease, and the results have been staggering. Proclaimed the fourth pillar of cancer therapy, immunotherapy has spawned complete remissions and responses for otherwise terminal cancer patients as well as been implicated in the fight against infectious and autoimmune diseases. Immunotherapies can target disease-associated antigens and prime the immune system to eradicate the malicious cells or species. Without specific targets, immunotherapies are frequently limited in their ability to aid the immune system. By enabling the identification of disease-related peptides and metabolites, MS allows us to develop immunotherapies against a meaningful target. The discovery of biomarkers benefits patients two-fold, as the biomarkers can be used both in diagnostics and for therapeutics. As discussed in our review, MS has garnered a wide array of biomarkers relevant to many diseases and paves the way for enhanced design of immunotherapies. MS has enabled the identification of multiple biomarkers of disease, from neoantigens that create personalized T cell therapies and cancer vaccines, to key molecules that play an influential role in *Salmonella* and *Francisella* infection, to pro-tumorigenic properties of cancer associated fibroblasts, and finally to peptides that can be used for the early detection of cardiovascular disease. Cardiovascular disease and cancer are the leading causes of death in the United States, and MS's ability to discern the peptides that facilitate these diseases will lead to improved diagnostic and therapeutic methods.

Although MS has unearthed a significant amount of high impact data, there is still much to learn. It is anticipated that every infectious disease has specific peptides that promote pathogenesis and every cancer has antigens that promotes its tumorigenesis. It is likely that every disease has specific biomarkers, but it is also important to consider that each individual's disease and immune response are different. Immunotherapy has altered the disease treatment landscape, but still faces the limitation of being efficacious in a fraction of patients. MS can provide the information needed for personalized medicine as well as widespread treatments through the discovery of universal antigens. Until a reduction in processing and equipment costs materializes, it is likely that personalized methods will not be economically feasible, which promotes a focus on recognizing ubiquitous biomarkers. The identification of biomarkers that can be used reliably will revolutionize diagnostics and lead to the development of generally usable immunotherapies. In addition to the discovery of biomarkers, the identification of the critical biomolecules involved in pathogenesis will lead to novel, highly effective therapies. For example, MS has discovered NNMT as the biomolecule essential to the promotion of cancer progression by CAFs, representing a shift from the previous emphasis on FAP. MS highlights the biomarkers and key biomolecules in diseases and in doing so elucidates the mechanisms and pathways of disease. As more labs have access to MS instrumentation and the computational databases used for protein analysis continue to improve, the number of disease components characterized will multiply. In order to formulate enhanced theranostic immunotherapies, we must gain a better understanding of disease and be able to identify disease, which can be accomplished by MS.

## Figures and Tables

**Tables 1:** Type of mass analyzers

Mass Analyzer	Quadrupole	Time of Flight	Orbitrap
Function	Applies an electrostatic field to force ion oscillation and then selecting only ions of a specific m/z range to pass to the detector	Ions are pushed through the analyzer with the same kinetic energy, causing them to reach the analyzer at a speed corresponding directly to their m/z ratio	Excites ions by pulsing radiofrequency signal through electrostatic field and then processes the signals generated by the ions using Fourier transform to obtain component frequency of the ions which correspond to their m/z ratio
Uses	LC-MS, LC-MS/MS, Triple Quad, Targeted	LC-MS, LC-MS/MS Triple TOF, SWATH	LC-MS/MS
Advantages	Can be used in tandem or on their own Highly sensitive and specific Functions as a mass filter	Relatively fast Relatively inexpensive Wide mass range	High resolution and accuracy Highly sensitive and specific Functions as a mass filter Wide mass range
Disadvantages	Limited to low mass range	Lower sensitivity and specificity	Available only as MS/MS instrument commercially Relatively expensive

## Abbreviations

ACR: albumin creatinine ratio
AML: acute myeloid leukemia
APC: antigen-presenting cells
BCAA: branched chain amino acids
CAF: cancer-associated fibroblast
CML: chronic myeloid leukemia
CSF: cerebrospinal fluid
DDA: data-dependent acquisition
DIA: data-independent acquisition
DIMS-MS: differential ion mobility spectrometry
eGFR: estimated glomerular filtration rate
ESI: Electrospray Ionization
FAP: fibroblast activating protein
FC: fold-change
FT-ICR: Fourier transform ion-cyclotron resonance
GC: gas chromatography
GVHD: graft-versus-host-disease
GVL: graft-versus-leukemia
HF: heart failure
HLA: human leukocyte antigen
HPLC: high performance liquid chromatography
ICP: inductively coupled plasma
IHC: immunohistochemistry
LC: liquid chromatography
LPS: lipopolysaccharides
m/z: mass-to-charge
MALDI: Matrix Assisted Laser Desorption/Ionization
MDS: myelodysplastic syndrome
MHC: major histocompatibility complex
MM: multiple myeloma
MPN: myeloproliferative neoplasms
MRD: minimal residual disease
MRM: multiple-reaction monitoring
MS/MS: tandem mass spectrometry
MS: mass spectrometry
MSI: mass spectrometry imaging
NGS: next generation sequencing
NNMT: nicotinamide N-methyltransferase
PBMCs: peripheral blood mononuclear cells
PD-1: programmed cell death-1
PD-L1: programmed cell death-1 ligand-1
PD-L2: programmed cell death-2 ligand 2
PMI: planned myocardial infarction
PRM: parallel-reaction monitoring
RA: Rheumatoid arthritis
SID: stable isotope dilution
TMAO: Trimethylamine N-oxide
TME: tumor microenvironment
TNF: tumor necrosis factor
TOF: time-of-flight
VDJ: variable, diversity, and joining gene
